# Bai-Hu-Jia-Ren-Shen-Tang Decoction Reduces Fatty Liver by Activating AMP-Activated Protein Kinase In Vitro and In Vivo

**DOI:** 10.1155/2015/651734

**Published:** 2015-10-05

**Authors:** Hui-Kang Liu, Tzu-Min Hung, Hsiu-Chen Huang, I-Jung Lee, Chia-Chuan Chang, Jing-Jy Cheng, Lie-Chwen Lin, Cheng Huang

**Affiliations:** ^1^National Research Institute of Chinese Medicine, Taipei 11221, Taiwan; ^2^Ph.D. Program for the Clinical Drug Discovery from Botanical Herbs, Taipei Medical University, Taipei 11031, Taiwan; ^3^Department of Medical Research, E-DA Hospital, Kaohsiung 82445, Taiwan; ^4^Department of Applied Science, National Hsinchu University of Education, Hsinchu 30014, Taiwan; ^5^School of Pharmacy, College of Medicine, National Taiwan University, Taipei 10051, Taiwan

## Abstract

Obesity and associated conditions, such as type 2 diabetes mellitus (T2DM) and nonalcoholic fatty liver disease (NAFLD), are currently a worldwide health problem. In Asian traditional medicine, Bai-Hu-Jia-Ren-Shen-Tang (BHJRST) is widely used in diabetes patients to reduce thirst. However, whether it has a therapeutic effect on T2DM or NAFLD is not known. The aim of this study was to examine whether BHJRST had a lipid-lowering effect using a HuS-E/2 cell model of fatty liver induced by palmitate and in a db/db mouse model of dyslipidemia. Incubation of HuS-E/2 cells with palmitate markedly increased lipid accumulation and expression of adipose triglyceride lipase (ATGL), which is involved in lipolysis. BHJRST significantly decreased lipid accumulation and increased ATGL levels and phosphorylation of AMP-activated protein kinase (AMPK) and its primary downstream target, acetyl-CoA carboxylase (ACC), which are involved in fatty acid oxidation. Furthermore, after twice daily oral administration for six weeks, BHJRST significantly reduced hepatic fat accumulation in db/db mice, as demonstrated by increased hepatic AMPK and ACC phosphorylation, reduced serum triglyceride levels, and reduced hepatic total lipid content. The results show that BHJRST has a lipid-lowering effect in the liver that is mediated by activation of the AMPK signaling pathway.

## 1. Introduction

Obesity and dysregulated insulin action in the liver are strongly associated and are currently a worldwide health problem [1]. Fatty liver, the initial stage of nonalcoholic fatty liver disease (NAFLD), is a common metabolic symptom [2] and is caused by an imbalance of lipid metabolism. NAFLD and type 2 diabetes mellitus (T2DM) frequently coexist, as they share the pathogenic abnormalities of excess adiposity and insulin resistance [3, 4]. Although the molecular mechanisms underlying fatty liver are not fully understood, dysregulation of hepatic lipid homeostasis caused by pathological conditions, such as reduced fatty acid oxidation, enhanced de novo lipogenesis, elevated hepatic fatty acid influx, and/or increased systemic insulin resistance, is thought to be important in its development [5]. Indeed, current therapies for fatty liver disease are aimed at reducing body weight and improving insulin sensitivity to alleviate the associated metabolic syndrome [6, 7].

The pathologic findings in NAFLD include accumulation of intracellular triglyceride in the parenchyma of the liver [8, 9]. Adipose triglyceride lipase (ATGL) and hormone-sensitive lipase (HSL) are the major triglyceride lipases in many tissues [10] and expression of both is decreased in the obese, insulin-resistant state, suggesting that insulin resistance is associated with impaired lipolysis [11, 12]. A large body of evidence indicates that AMP-activated protein kinase (AMPK) is important in regulating hepatic lipogenesis [13]. In the liver, activation of AMPK by phosphorylation of threonine 172 switches off fatty acid synthesis by increasing the phosphorylation and inactivation of acetyl-CoA carboxylase (ACC) [14]. Some antidiabetic drugs, such as metformin and the thiazolidinediones, alleviate fatty liver in humans and rodents by downregulating lipid metabolism through AMPK activation [15]. Thus, AMPK represents an attractive target for therapeutic intervention in the treatment of fatty liver disorders [16, 17].

Bai-Hu-Tang (BHT), composed of Gypsum Fibrosum (Shi-Gao), Rhizoma Anemarrhenae (Zhi-Mu), Radix Glycyrrhizae Preparata (Zhi-Gan-Cao), and seed of* Oryza sativa* (Jing-Mi), is a traditional Chinese medicine described in the Chinese medicine book “Discussion of Cold Damage” (“Shang-Han-Lun” in Chinese), which has been used in China for over 1800 years. BHT potentiates insulin-stimulated glucose uptake in vitro [18]. The formula used in this study was Bai-Hu-Jia-Ren-Shen-Tang (BHJRST), which is an enhanced formula of BHT prepared by addition of Ginseng Radix (Ren-Shen). Traditionally, BHJRST is used to reduce thirst in diabetes patients and is the most common herbal formula prescribed by traditional Chinese medicine doctors for the treatment of type 2 diabetes mellitus (T2DM) in Taiwan [19]. Although BHJRST has been reported to have significant antihyperglycemic activity [20], it is not known whether it has a fat-lowering action.

In this study, we used a human hepatic cell line and an animal model to investigate the mechanisms responsible for the in vitro and in vivo effects of BHJRST on lipid metabolism. In the in vitro study, immortalized primary human hepatocytes, HuS-E/2 cells [21], which are phenotypically and functionally similar to primary hepatocytes, were used to established a fatty liver cell model. In in vivo experiments, db/db mice, which show dyslipidemia similar to that seen in patients with T2DM, were used as a model to study the pathogenesis and treatment of diabetic dyslipidemia [22].

## 2. Materials and Methods

### 2.1. Preparation of the BHJRST Formula and Single Remedy Extracts

Rhizome of Rhizoma Anemarrhenae (Zhi-Mu), Gypsum Fibrosum (Shi-Gao), root of Radix Glycyrrhizae Preparata (Zhi-Gan-Cao), seed of* Oryza sativa* (Jing-Mi), and root of Ginseng Radix (Ren-Shen) were mixed in a classical dosage ratio used in the Han Dynasty (6 parts by weight of Zhimu, 16 parts Shigao, 2 parts Gancao, 8 parts Jingmi, and 3 parts Renshen). To prepare the water extracts, 35 g of the mixture or the corresponding weight of each single ingredient (e.g., 6 g of Zhimu) was added to 400 mL of water and refluxed at 100°C for 1 h; then, the supernatant was collected, clarified by centrifugation at 1000 g for 10 min at 4°C, and either used directly as such in animal experiments or lyophilized and dissolved in DMSO for cell studies.

### 2.2. Confirmation of the Identity of the Ingredients by Microscopy

All the specimens were sliced manually by hand, fixed in a solution of 50% glycerin in water, and observed under a microscope (Carl Zeiss Inc., Germany) to confirm their identity.

### 2.3. HPLC Sample Preparation

Each lyophilized sample (5 mg) was dissolved in 5 mL of DMSO, ultrasonicated for 30 s, and then injected into solid-phase extraction tubes (Strata-X 33 *μ*m Polymeric Reversed Phase, 200 mg/6 mL) (Phenomenex), which had been activated in advance by MeOH and equilibrated in distilled water. The tubes were then washed with distilled water, and the adsorbed materials were eluted with 10 mL of MeOH and filtered on a 0.45 *μ*m filter (13 mm, Millex-GV, EMD Millipore) for HPLC analysis.

### 2.4. Reverse-Phase HPLC Chemical Fingerprint Analysis of BHJRST

All HPLC fingerprint analyses of the water extracts of BHJRST and the single remedies of BHJRST were performed on a HPLC instrument equipped with a Shimadzu 10A system controller (both from Shimadzu Corporation, Kyoto, Japan). The injection volume was 10 *μ*L, the column was a Nacalai Cosmosil 5C_18_-AR-II Waters (5 *μ*m, 4.6 × 250 mm; Nacalai Tesque), the linear gradient was 10.0–90.0% B (A = MeOH, B = H_2_O) in 60 min, detection was at 280 nm, and the flow rate was 0.8 mL/min.

### 2.5. Antibodies and Chemical Reagents

Antibodies against ATGL, AMPK, pACC (Ser 79), ACC, tubulin, or actin were obtained from Genetex, the anti-pAMPK (Thr 172) antibodies were from Millipore, and the horseradish peroxidase-conjugated anti-mouse or anti-rabbit IgG antibodies were from Jackson ImmunoResearch Laboratories Inc. Palmitate and Oil Red O were purchased from Sigma.

### 2.6. Cell Culture and Treatment

HuS-E/2 cells, kindly provided by Dr. Shimotohno (Kyoto University, Japan), were maintained as described previously in primary hepatocyte medium (PH medium) [DMEM with 25 mM glucose (Gibco) containing 20 mM HEPES, 10% fetal bovine serum, 15 *μ*g/mL of L-proline, 0.25 *μ*g/mL of insulin, 5 × 10^−8 ^M dexamethasone, 44 mM NaHCO_3_, 10 mM nicotinamide, 5 ng/mL of EGF, 0.1 mM Asc-2P, 100 IU/mL of penicillin, 100 *μ*g/mL of streptomycin, 10 *μ*g/mL of gentamicin, and 1 *μ*g/mL of plasmocin] [21]. To induce fatty acid overload, HuS-E/2 cells at 70% confluence were cultured in glucose-free PH medium [made as above, but with glucose-free DMEM (Sigma)] and incubated with the indicated concentration of palmitate for 24 h. The palmitate used was provided in the form of a palmitate/BSA complex prepared as described previously [23]. To study the effect of BHJRST, the cells were incubated with various concentrations of palmitate and either BHJRST or a final concentration of 0.1% DMSO (vehicle) for the indicated time.

### 2.7. Cell Viability Assay

Cell viability was assessed using the 3-(4,5-dimethylthiazol-2-yl)-2,5-diphenyltetrazolium bromide (MTT) assay. MTT assay performed according to the manufacturer's suggestions (Sigma). HuS-E/2 cells were added to 96-well plates at a density of 1 × 10^4^ cells per well in 100 *μ*L of PH medium and allowed to attach for 18 h; then, the medium was changed to glucose-free PH medium containing different concentrations of BHJRST or 0.1% DMSO (vehicle control) and 0.1 mM palmitate for 24 h. They were then incubated with MTT for another 4 h at 37°C; then, the medium was removed and 100 *μ*L of DMSO was added to each well. The absorbance of the samples was measured at 570 nm using a FlexStation 3 microplate reader (Molecular Devices).

### 2.8. Oil Red O Staining

To measure intracellular lipid content, HuS-E/2 cells in 6-well plates were stained using the Oil Red O method [24]. Briefly, the cells were fixed in 4% paraformaldehyde in phosphate-buffered saline (PBS) for 30 min at room temperature (23°C), stained with Oil Red O (stock solution, 3 mg/mL in isopropanol; working solution, 60% Oil Red O stock solution and 40% distilled water) for 1 h at room temperature, and then rinsed with water, and images were captured under a microscope. For quantitative analysis of cellular lipids, the cells were washed three times with ice-cold PBS, fixed with 10% formalin for 1 h, washed, stained with Oil Red O solution for 1 h at room temperature, and washed with water to remove nonbound dye; then, 1 mL of isopropanol was added to each well and the plate was shaken at room temperature for 5 min; then, the absorbance at 510 nm was read on a spectrophotometer.

### 2.9. Western Blot Analysis

After treatment, HuS-E/2 cells were harvested in lysis buffer (50 mM Tris-HCl, pH 8.0, 5 mM EDTA, 150 mM NaCl, 0.5% Nonidet P-40, 0.5 mM phenylmethylsulfonyl fluoride, and 0.5 mM dithiothreitol) and incubated for 30 min at 4°C; then, the samples were sonicated for 3 × 5 s with 15 s breaks and centrifuged at 12000 g for 10 min at 4°C. The protein concentrations of the supernatants were determined using a protein assay kit (Bio-Rad); then, equal amounts of total cellular protein (200 mg) were resolved by SDS-PAGE, transferred onto polyvinylidene difluoride membranes (Amersham Biosciences), and probed with primary antibody, followed by horseradish peroxidase-conjugated secondary antibody; then, bound antibody was visualized using enhanced chemiluminescence kits (Amersham Biosciences).

### 2.10. Total Lipid, Triglyceride, and Cholesterol Assay

For lipid determinations, cell homogenate, mouse serum, or mouse liver was extracted using a modified Bligh and Dyer procedure [25]. In brief, the sample was homogenized at room temperature with a mixture of chloroform-methanol-water (8 : 4 : 3) and the resulting mixture was shaken at 37°C for 1 h and then centrifuged at 1,000 g for 20 min at 4°C, and the supernatant was collected for lipid analysis. Triacylglycerol, total cholesterol, and total lipid levels were measured using enzymatic method kits from Randox Laboratories in accordance with the manufacturer's instructions.

### 2.11. Animal Experiments

Six- to 8-week-old male BKS.Cg-Lepr^db^+/+Lepr^db^/Jnarl (db/db) mice were purchased from The National Laboratory Animal Center, Taipei, Taiwan, and were housed at room temperature with controlled humidity and on a 12 h/12 h light/dark cycle (lights on at 7.00 a.m.) at the Animal Center of the National Research Institute of Chinese Medicine (NRICM), Taipei, Taiwan. The use of animals for this research was approved by the Animal Research Committee of the NRICM and all procedures followed The Guide for the Care and Use of Laboratory Animals (NIH publication, 85-23, revised 1996) and the guidelines of the Animal Welfare Act, Taiwan.

The mice were fed a standard diet (7.9% moisture, 22.9% crude protein, 5.4% crude fat, 6.2% crude ash, 3.4% crude fiber, and 54.2% nitrogen-free extract; Oriental Yeast Co., Ltd. data sheet) throughout the study, but, at the age of 12 weeks, they were divided into two groups which received either double-distilled water or BHJRST (900 mg/kg b.w. in double-distilled water) twice daily by gavage for 6 weeks. At 18 weeks, serum samples were collected prior to sacrifice and the liver was harvested for protein and lipid analysis.

### 2.12. Statistical Analysis

All values are expressed as the mean ± SD of the results from at least three separate experiments. One-way ANOVA followed by Dunnett's multiple comparison test was used to compare differences among groups of samples. Asterisks indicate that the values were significantly different from the control (^*∗*^ *p* < 0.05; ^*∗∗*^ *p* < 0.01).

## 3. Results

### 3.1. Characterization of BHJRST

**Figure 1:**
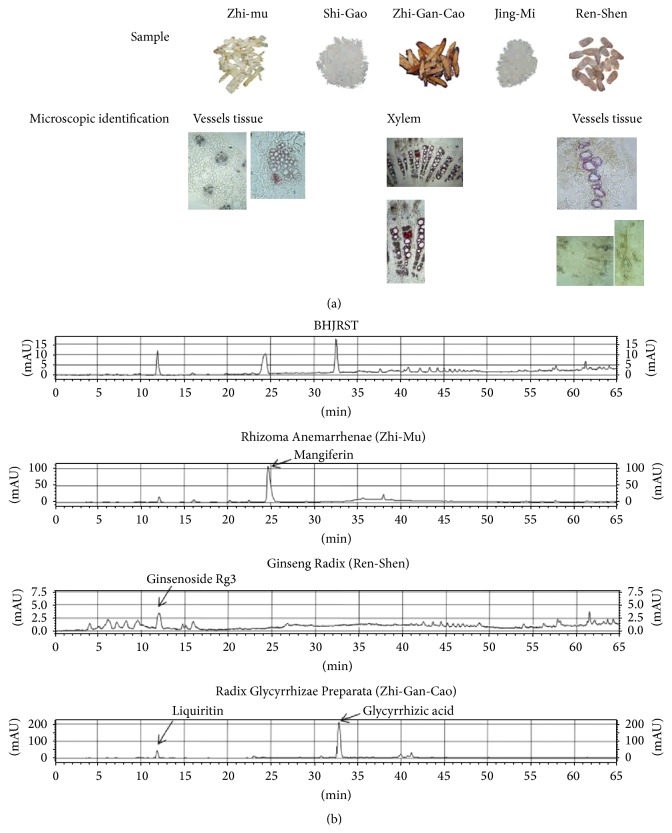
Characterization of BHJRST. (a) Confirmation of the identity of the ingredients. The macroscopic and microscopic appearance of the 5 ingredients used to prepare BHJRST was examined. (b) HPLC chromatograms of water extracts of BHJRST and three of the five single ingredients. Three major peaks were identified in the classical BHJRST formula (top panel) by comparison of their retention times with those of peaks in extracts of the single ingredients Rhizoma Anemarrhenae (Zhi-Mu), Ginseng Radix (Ren-Shen), or Radix Glycyrrhizae Preparata (Zhi-Gan-Cao).

Ginseng-plus-Bai-Hu-Tang (BHJRST) is composed of five crude ingredients and the appearance and microscopic features of each of these were examined to confirm the identity of the ingredient (Figure 1(a)). HPLC pattern analysis, the so-called “fingerprint” method, was performed on the water extracts of BHJRST and three of the single ingredients (Figure 1(b)); analysis of the other two single ingredients, Gypsum Fibrosum (Shi-Gao) and seed of* Oryza sativa* (Jing-Mi), was not performed, as their active components are believed to be, respectively, inorganic (CaSO_4_) or macromolecular (polysaccharides). The HPLC chromatogram of the extract prepared from BHJRST (Figure 1(b), top panel) showed three major peaks at 11.9, 24.4, and 32.6 min, corresponding to ginsenoside Rg3 from Ginseng Radix (Ren-Shen) (12.0 min), mangiferin from Rhizoma Anemarrhenae (Zhi-Mu) (24.6 min), and glycyrrhizic acid from Radix Glycyrrhizae Preparata (Zhi-Gan-Cao) (32.7 min).

### 3.2. A Fatty Liver Cell Model: A High Fat Environment Increases Intracellular Lipid Formation in HuS-E/2 Cells and Induces ATGL Expression

**Figure 2:**
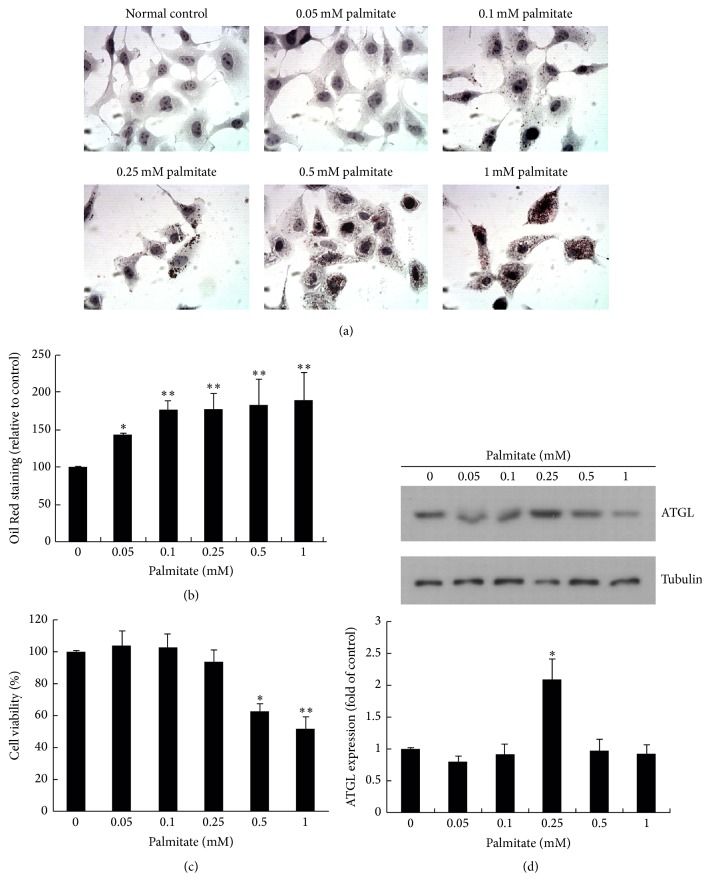
The fatty liver cell model—a high fat environment increases intracellular lipid formation in HuS-E/2 cells and induces ATGL expression. (a) Micrographs of HuS-E/2 cells incubated for 24 h in glucose-free PH medium with different concentrations of palmitate to simulate a high fat environment. Cells were stained with Oil Red O and observed under a microscope at 400x original magnification. (b) Quantitative analysis of lipid deposition in cells using Oil Red O staining. HuS-E/2 cells were treated with the indicated concentration of palmitate in glucose-free PH medium for 24 h. The data represent the mean ± SD for three independent experiments and are expressed as a percentage of the control value. (c) Cell viability in the high fat environment. HuS-E/2 cells were incubated with the indicated concentration of palmitate in glucose-free PH medium for 24 h as above; then, cell viability was determined using the MTT assay. The data represent the mean ± SD for three independent experiments and are expressed as a percentage of the control value. (d) Western blots showing ATGL and tubulin levels in HuS-E/2 cells incubated with the indicated concentration of palmitate in glucose-free PH medium for 24 h. The upper panel shows a representative result of those obtained in three experiments and the lower panel shows the quantitative analysis of ATGL expression normalized to that of tubulin and expressed as a fold value relative to the control value. The data represent the mean ± SD for three independent experiments. ^*∗*^ *p* < 0.05 and ^*∗∗*^ *p* < 0.01 versus control.

Fatty liver disease is mainly due to triglyceride accumulation in hepatocytes [26]. To determine whether liver cells esterify and deposit fatty acid as lipid droplets, HuS-E/2 immortalized human primary hepatocytes were incubated in glucose-free PH medium alone or containing 0.05–1 mM palmitate; then, intracellular lipid accumulation was measured using oil Red O staining. The results (Figures 2(a) and 2(b)) showed that HuS-E/2 cells exposed to palmitate showed a clear dose-dependent increase in lipid accumulation in the cytosol compared to the control, indicating that a cell model of steatosis was induced by palmitate. Incubation of HuS-E/2 cells with concentrations of palmitate lower than 0.25 mM did not affect cell viability, as demonstrated by the MTT assay (Figure 2(c)). Since ATGL is responsible for the catabolism of cellular lipid stores [12] and since we wanted conditions in which lipid could accumulate in the absence of ATGL overexpression, ATGL expression was examined by western blotting in HuS-E/2 cells treated as above. As shown in Figure 2(d), ATGL expression was low when cells were incubated in glucose-free PH medium alone and was increased by treatment of cells with 0.25 mM palmitate for 24 h, but not by treatment with 0.1 mM palmitate. Thus, we established the parameters for a fatty liver cell model by incubating HuS-E/2 cells in high fat (0.1 mM palmitate) glucose-free PH medium.

### 3.3. Effect of BHJRST or Palmitate on Cell Survival

**Figure 3:**
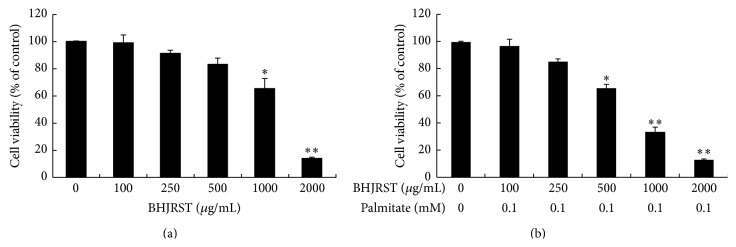
Effects of BHJRST on cell survival. Cells were cultured for 24 h in the presence of the indicated concentration of BHJRST alone (a) or together with 0 or 0.1 mM palmitate (b) in glucose-free PH medium; then, cell viability was measured using the MTT assay and expressed as a percentage of the value for untreated cells. The results are the mean ± SD for three independent experiments. ^*∗*^ *p* < 0.05 and ^*∗∗*^ *p* < 0.01 versus control.

To examine the effect of BHJRST on cell viability, HuS-E/2 cells were incubated with glucose-free PH medium alone or containing 100–2000 *μ*g/mL of BHJRST with or without 0.1 mM palmitate then an MTT assay was performed. As shown in Figure 3(a), BHJRST concentrations of 100, 250, or 500 *μ*g/mL alone had no significant effect on viability, but a significant decrease was seen using concentrations of 1000 and 2000 *μ*g/mL. Palmitate (0.1 mM) alone or in combination with 100 or 250 *μ*g/mL of BHJRST also had no cytotoxic effect (Figure 3(b)). BHJRST concentrations of 100 and 250 *μ*g/mL were therefore used in subsequent studies.

### 3.4. BHJRST Inhibits Palmitate-Induced Cellular Lipid Accumulation

**Figure 4:**
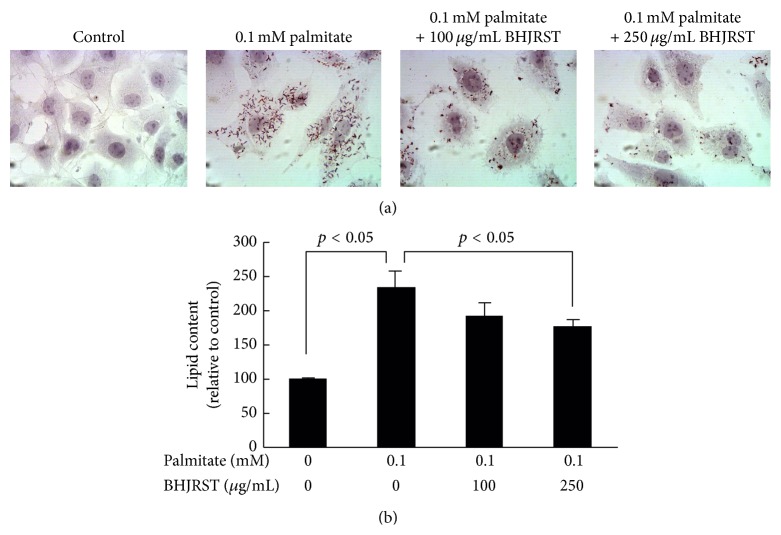
BHJRST inhibits palmitate-induced cellular lipid accumulation. (a) HuS-E/2 cells were treated with the indicated concentrations of BHJRST and palmitate in glucose-free PH medium for 24 h; then, images of the Oil Red O-stained cells were captured using a microscope at 400x original magnification. (b) HuS-E/2 cells were incubated for 24 h with the indicated concentrations of BHJRST and palmitate as above; then, quantitative analysis of lipid deposition in the Oil Red O-stained cells was performed. The data represent the mean ± SD for three independent experiments.

To examine the ability of BHJRST to inhibit palmitate-induced lipid accumulation, HuS-E/2 cells were incubated in glucose-free PH medium alone or containing 0.1 mM palmitate with or without 100 or 250 *μ*g/mL of BHJRST, then total lipid levels were measured by Oil Red O staining. As shown in Figure 4(a), treatment with 250 *μ*g/mL of BHJRST significantly reduced palmitate-induced cellular lipid accumulation. These results were confirmed by quantification of the intracellular lipid content (Figure 4(b)).

### 3.5. BHJRST Stimulates AMPK Phosphorylation and ATGL Expression under High Fat Conditions

**Figure 5:**
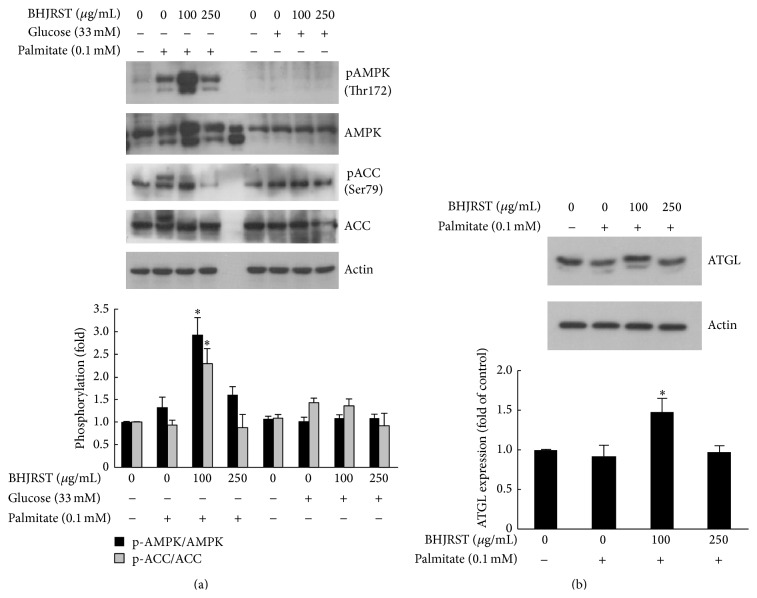
BHJRST stimulates AMPK phosphorylation and ATGL expression under high fat conditions. (a) HuS-E/2 cells were left untreated or were incubated for 24 h with or without 0.1 mM palmitate in glucose-free PH medium with or without BHJRST (100 or 250 *μ*g/mL) (left section) or in PH medium containing 33 mM glucose with or without BHJRST (100 or 250 *μ*g/mL) (right section); then, they were analyzed for phosphorylation of AMPK at Thr172 and ACC at Ser-79, total AMPK and ACC, and actin. Representative immunoblots are shown in the upper panel and the densitometric analysis of AMPK and ACC phosphorylation is shown in the lower panel; the results are the mean ± SD for three independent experiments for the intensity of the phosphorylated band divided by that for the “total” band expressed as a fold value of the control value. (b) Western blot analysis of the expression of ATGL and actin in untreated HuS-E/2 cells and cells incubated for 24 h with 0.1 mM palmitate and 0, 100, or 250 *μ*g/mL of BHJRST as above. The upper panel shows a representative blot and the lower panel shows the quantitative analysis of ATGL expression normalized to actin levels and expressed as a fold value compared to the control value. The data represent the mean ± SD for three independent experiments. ^*∗*^ *p* < 0.05 versus control.

Having shown that BHJRST had an inhibitory effect on the palmitate-induced increase in hepatocyte lipid levels, the possible mechanisms responsible for this effect were assessed. Since AMPK responds to changes in cellular energy status [14] and has been suggested to play a crucial role in regulating fat metabolism in the liver, the effect of BHJRST on AMPK activation in HuS-E/2 cells was examined. Activation of AMPK, which correlates tightly with phosphorylation at Thr-172, and inactivation of its primary downstream target enzyme ACC by phosphorylation at Ser-79 were assessed by measuring phosphorylation at these sites by western blotting. To determine whether BHJRST increased phospho-AMPK levels, cells were incubated in glucose-free PH medium alone or containing 0.1 mM palmitate in the presence or absence of 100 and 250 *μ*g/mL of BHJRST or in high glucose (33 mM) medium (glucose-free PH medium with 33 mM glucose added) in the presence or absence of 100 and 250 *μ*g/mL of BHJRST. As shown in Figure 5(a), 100 *μ*g/mL, but not 250 *μ*g/mL, of BHJRST significantly increased AMPK phosphorylation at Thr-172 in fatty acid-overloaded HuS-E/2 cells, but not in high glucose-treated cells, and the increased AMPK phosphorylation was accompanied by a significant increase in ACC phosphorylation at Ser-79, indicating that BHJRST-induced activation of AMPK led to inhibition of ACC.

To determine whether BHJRST also induced ATGL expression in the high fat condition, cells were incubated in glucose-free PH medium alone or containing 0.1 mM palmitate with or without 100 or 250 *μ*g/mL of BHJRST. As shown in Figure 5(b), treatment with 100 *μ*g/mL, but not 250 *μ*g/mL, of BHJRST increased ATGL protein levels in HuS-E/2 cells. These results show that, in vitro, AMPK activation by BHJRST increases ATGL expression under high fat conditions.

### 3.6. BHJRST Treatment Stimulates AMPK Phosphorylation and Reduces Liver Lipid Accumulation in db/db Mice

**Figure 6:**
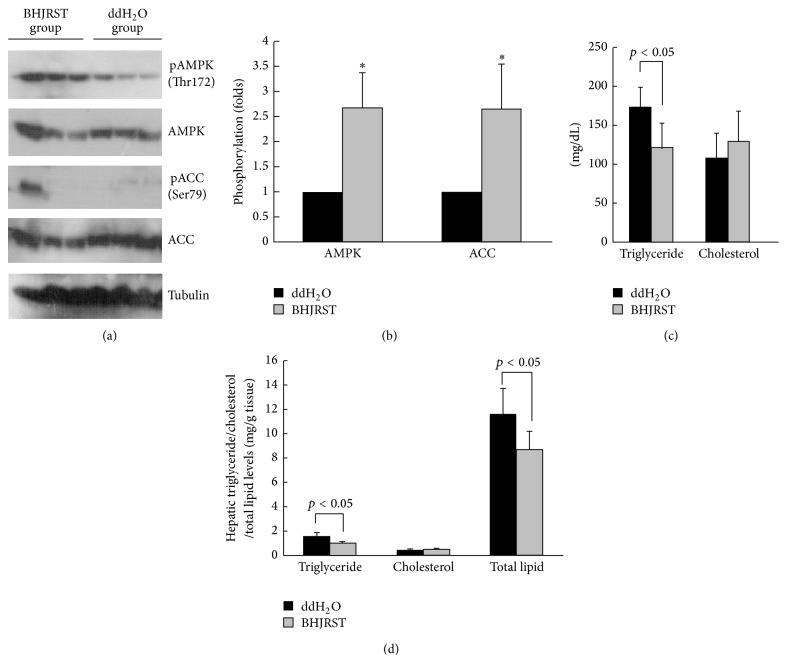
BHJRST treatment of db/db mice stimulates AMPK phosphorylation and reduces liver lipid accumulation. Animals (5 per group) were given BHJRST or double-distilled water (*n* = 5) twice daily by gavage for 6 weeks; then, they were euthanized for analysis of liver tissues and serum, as described in Section 2. (a) Representative immunoblots of liver for phosphorylation for AMPK and ACC, total AMPK and ACC, and tubulin. (b) Densitometric analysis of phosphorylation of AMPK and ACC levels. The results are the mean ± SD for the intensity of the phosphorylated band divided by that for the “total” band expressed as a fold value of the control value. (c) Serum triglyceride and cholesterol levels. The data represent the mean ± SD. (d) Hepatic triglyceride, cholesterol, and total lipid levels. The data represent the mean ± SD. In (b–d), the *p* value compared to the control group is shown either as a value or as ^*∗*^ *p* < 0.05.

The db/db mouse has high plasma levels of triglyceride and cholesterol and is a good model for diabetic dyslipidemia [22]. To examine whether BHJRST treatment could prevent liver lipid accumulation in vivo, we administered BHJRST (900 mg BHJRST/kg) or double-distilled water (control) twice daily by gavage for 6 weeks to male db/db mice fed a standard diet. Consistent with the upregulated AMPK phosphorylation seen in vitro, as shown in Figures 6(a) and 6(b), BHJRST treatment resulted in significantly increased AMPK phosphorylation at The-172 in liver tissue lysates and also in significantly increased ACC phosphorylation at Ser-79. To test the effects of BHJRST on lipid homeostasis, we next measured serum and hepatic lipid levels.  Figure 6(c) shows that the BHJRST-treated group had significantly lower serum levels of triglyceride compared to the controls, while Figure 6(d) shows that BHJRST administration also resulted in decreases of 40.4% in hepatic triglyceride levels and 25.5% in hepatic total lipid levels. Together, these data suggest that BHJRST induces activation of AMPK, which translates into inhibition of ACC and leads to a decrease in hepatic fatty acid synthesis and lipid accumulation.

## 4. Discussion

Fatty liver is characterized by increased levels of hepatocellular lipids and is frequently associated with steatohepatitis and hepatocellular injury, which eventually can result in severe liver damage, including bridging fibrosis and cirrhosis [27]. Current treatment strategies aim to improve insulin resistance by weight loss and exercise, improving insulin sensitivity by the use of insulin-sensitizing agents (e.g., pioglitazone) and reducing oxidative stress by the use of antioxidants, such as vitamin E. Some Chinese medicines with hypolipidemic, antidiabetic, and antiobesity effects have been used by traditional Chinese medicine doctors for over a thousand years. However, their therapeutic mechanism remains unclear. Bai-Hu-Jia-Ren-Shen-Tang (BHJRST) is one of the most common herbal medicines used to treat T2DM patients in Taiwan [19]. These findings prompted us to ask whether BHJRST could reduce the hepatic fat accumulation associated with obesity and to determine the mechanisms responsible for the therapeutic effect of BHJRST in fatty liver disease. Here, we report the new finding that BHRST inhibits cellular lipid accumulation through activation of AMPK. In the liver, the AMPK complex, an evolutionally conserved serine/threonine heterotrimer kinase complex [28], is emerging as a possible target molecule for antiobesity therapy, as its activation results in increased fatty acid oxidation and decreased lipid synthesis [29]. In addition, inactivation of ACC reduces the synthesis of malonyl-CoA and activates fatty acid oxidation [30].

BHJRST is an enhanced formula in which Ginseng Radix (Ren-Shen) is added to the Bai-Hu-Tang (BHT) formula. Traditionally, BHJRST, but not BHT, is used to decrease thirst in diabetes patients. In a recent study, a water extract of ginseng root was found to have a fat-lowering action in vivo [31]. Ginsenoside Rb1 (Rb1), a compound extracted from ginseng root, has a glucose-lowering action in vitro [32] and significantly reduces body weight, improves glucose tolerance, and enhances insulin action in high fat diet-induced obese rats [33]. In our study, we found that if the content of Gypsum Fibrosum (Shi-Gao) in BHJRST was reduced from 16 g to 10 g, the concentrations of ginsenoside Rg3 and mangiferin in the final extract were reduced, as shown by HPLC analysis (data not shown), suggesting that Gypsum Fibrosum (Shi-Gao) may help in dissolving these ingredients.

Normal human hepatocytes are the ideal system in which to study the liver-specific metabolism of lipid, but when cultured in vitro, they proliferate poorly and divide only a few times. The most common cell line used to study liver lipid metabolism is the hepatoma-derived HepG2 cell line, but there is concern about its use, as it was derived from liver tissue with differentiated hepatocellular carcinoma and is probably genetically distinct from primary hepatocytes. In this study, we used the HuS-E/2 cell line, which was derived from normal hepatocytes and has been shown to be phenotypically and functionally similar to primary hepatocytes [21, 34]. We investigated the ability of BHJRST to prevent fat deposition using an HuS-E/2 cell model of fatty liver induced by palmitate and found that 0.1 mM palmitate resulted in marked fat accumulation, as demonstrated by Oil Red O staining (Figure 2), and that coaddition of 100 *μ*g/mL of BHJRST significantly reduced the amount of accumulated lipid (Figure 4), without having a cytotoxic effect (Figure 3). In addition, treatment with 100 *μ*g/mL of BHJRST increased phosphorylation of AMPK and ACC and upregulated ATGL protein expression in HuS-E/2 cells (Figure 5). Thus, we demonstrated that HuS-E/2 cells develop palmitate-induced hepatic lipid accumulation and that BHJRST has a protective effect associated with a significant increase in hepatic AMPK activation and hepatic ATGL expression.

Animal models of fatty liver disease can arise as a result of induced genetic mutation and most published studies have employed the leptin-resistant (db/db) mouse [35]. In this study, we used db/db mice and showed that BHJRST also activated AMPK and reduced ACC activation and lipid levels in vivo (Figure 6). These results were confirmed by quantification of intracellular triglyceride and total lipid levels. We suggest that AMPK and ACC are the critical components involved in the BHJRST-induced inhibition of lipid synthesis in the db/db mice liver, implying a role of BHJRST in energy balance control by modulating lipid biosynthesis. It has been demonstrated that AMPK increases glucose transporter 4 (GLUT 4) transposition and GLUT 4 gene transcription in muscle and liver [36], suggesting a possible mechanism by which BHJRST modulates homoeostasis of lipid and carbohydrate metabolism in living cells.

ATGL is important for basal lipolysis and is responsible for the first step in the breakdown of fat. Regulation of ATGL expression is impaired with age and could contribute to the observed increased difficulty in metabolizing lipids [37]. It is the rate-limiting enzyme in triglyceride hydrolysis, which produces free fatty acids, which are released into the medium and do not accumulate in cells [10]. The findings agree with the fact that calorie restriction and exercise can be used to treat nonalcoholic fatty liver disease, as both activate AMPK. Our data are in agreement with the reports of Reid et al. and Caimari et al. showing that an increase in ATGL levels results in decreased hepatic lipogenesis and liver triglyceride accumulation and secretion.

Palmitate, a lipotoxic fatty acid, increases oxidative stress and ROS production and induces insulin resistance in hepatocytes [38]. Indeed, it has been shown that ROS levels are increased in patients and mice with clinical conditions associated with insulin resistance, such as obesity and T2DM, that palmitate treatment of cultured adipocytes induces cellular oxidative stress and ROS generation, and that a decrease in ROS production in obese mice contributes to a reduction in palmitate-induced lipid accumulation [39]. It will be very interesting to examine whether BHJRST is an antioxidant and can lower ROS production.

In summary, we have demonstrated that activation of the AMPK signaling pathway plays a critical role in the inhibitory effect of BHJRST on lipid metabolism in vitro and in vivo. These findings provide molecular evidence for the use of BHJRST as therapy for the management of fatty liver diseases. Unraveling the molecular mechanisms by which BHJRST controls energy balance by modulating lipid biosynthesis will provide new insights into the pathogenesis of these diseases and open avenues for novel therapeutic strategies.

## Abbreviations

ACC: Acetyl-CoA carboxylase
AMPK: AMP-activated protein kinase
ATGL: Adipose triglyceride lipase
BHJRST: Bai-Hu-Jia-Ren-Shen-Tang
NAFLD: Nonalcoholic fatty liver disease
PH medium: Primary hepatocyte medium
T2DM: Type 2 diabetes mellitus.
